# A Long-Distance RF-Powered Sensor Node with Adaptive Power Management for IoT Applications

**DOI:** 10.3390/s17081732

**Published:** 2017-07-28

**Authors:** Matteo Pizzotti, Luca Perilli, Massimo del Prete, Davide Fabbri, Roberto Canegallo, Michele Dini, Diego Masotti, Alessandra Costanzo, Eleonora Franchi Scarselli, Aldo Romani

**Affiliations:** 1 Advanced Research Center on Electronic Systems, University of Bologna, Via Toffano 2/2, Bologna 40126, Italy; luca.perilli@unibo.it (L.P.); michele.dini3@unibo.it (M.D.); alessandra.costanzo@unibo.it (A.C.); eleonora.franchi@unibo.it (E.F.S.); aldo.romani@unibo.it (A.R.); 2 Department of Electrical, Electronic, and Information Engineering, University of Bologna, Via Risorgimento 2, Bologna 40126, Italy; massimo.delprete3@unibo.it (M.d.P.); davide.fabbri21@unibo.it (D.F.); diego.masotti@unibo.it (D.M.); 3 STMicroelectronics, Via Camillo Olivetti, Agrate Brianza 20864, Italy; roberto.canegallo@st.com

**Keywords:** wireless sensor networks, RF power transfer, energy harvesting, nano-power DC/DC converter, rectifying antenna, ultra-low power sensor node, adaptive power management

## Abstract

We present a self-sustained battery-less multi-sensor platform with RF harvesting capability down to −17 dBm and implementing a standard DASH7 wireless communication interface. The node operates at distances up to 17 m from a 2 W UHF carrier. RF power transfer allows operation when common energy scavenging sources (e.g., sun, heat, etc.) are not available, while the DASH7 communication protocol makes it fully compatible with a standard IoT infrastructure. An optimized energy-harvesting module has been designed, including a rectifying antenna (rectenna) and an integrated nano-power DC/DC converter performing maximum-power-point-tracking (MPPT). A nonlinear/electromagnetic co-design procedure is adopted to design the rectenna, which is optimized to operate at ultra-low power levels. An ultra-low power microcontroller controls on-board sensors and wireless protocol, to adapt the power consumption to the available detected power by changing wake-up policies. As a result, adaptive behavior can be observed in the designed platform, to the extent that the transmission data rate is dynamically determined by RF power. Among the novel features of the system, we highlight the use of nano-power energy harvesting, the implementation of specific hardware/software wake-up policies, optimized algorithms for best sampling rate implementation, and adaptive behavior by the node based on the power received.

## 1. Introduction

Increasing interest in distributed sensor networks [1] and IoT applications has driven research into the scope of energy harvesting mechanisms towards a more precise field of application. The combination of smart nodes, able to interact with standard wireless communication infrastructures, and energy scavenging modules, which allow nodes to work in a standalone scenario, has proved crucial for the development of both technologies [2].

Since autonomous nodes are expected to operate in very dissimilar surroundings with different energy sources and power densities, the importance of finding efficient ways to exploit available energy is evident, as in many cases the power available from the environment is in the order of microwatts [3] or less. In this context, radio-frequency power harvesting [4,5] represents both a fascinating solution, due to the opportunity of selectively providing energy through dedicated RF energy showers augmented by smart power beaming techniques [6,7], and a tough challenge because of the limitations imposed by regulations causing very low voltage and power levels as the distance from the source increases. As a matter of fact, the most prohibitive obstacle to the diffusion of RF harvesting nodes is the lack of dedicated power converters able to operate under these conditions. However, simply developing specific power converters or designing more efficient rectennas is not sufficient: power management must go hand in hand not only with obvious requirements in terms of ultra-low power, but also with the development of specific policies for adjusting the behavior of the node according to the availability of energy. Active interaction between the power module and smart node is integral to achieving this target. Another important aspect that reinforces the need for such interaction is the considerable difference between the power profiles of the harvesting source and the active node; while the former can be considered constant or slowly changeable, the latter is characterized by short peak consumptions, followed by long inactive periods. This mismatch calls for the introduction of storage elements, e.g., capacitors or super-capacitors, which must be dimensioned carefully and considered as an integral part of optimization policies.

Thus, a holistic approach can best tackle this type of issue. On the one hand, circuit design aims at minimizing the power consumption of electronic devices and, on the other hand, optimized behavioral policies for active node modules need investigating with a view to exploiting the available energy at its best. The solution presented is designed from an ultra-low power approach, with the introduction of a custom rectenna, a dedicated nano-power DC/DC converter designed to operate down to 250 mV and 1 μW, and a multi-sensor node with a low-power profile and adopting a standard wireless communication protocol for IoT. In order to implement the necessary interactions between the power management subsection and the active sensor node, specific circuitry has been designed, dynamically changing the behavior of the system according to different scenarios of available environmental energy. The peculiar achievement obtained through such active interaction is the possibility to modulate the transmission data-rate as a consequence of variations in the power harvested, so that a higher amount of power causes an automatic increase in the data-rate and vice-versa, in such a way that the highest feasible data-rate is always obtained. Such an outcome may have a considerable rebound on those applications where high transmission rates are not strictly indispensable but can help in building a more extensive information database and therefore a more precise behavioral model for the monitored system.

**Figure 1:**
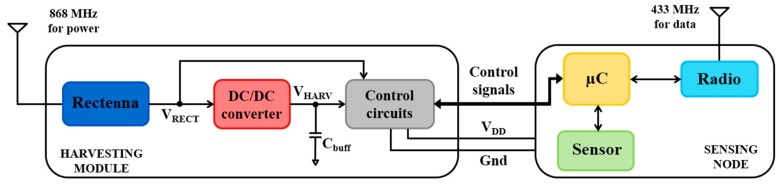
General scheme of the overall system.

In Figure 1 the overall system presented consists of two sub-systems: a harvesting module and a sensing node. The interaction presented is obtained through specific control signals exchanged between the power module and the microcontroller, designed to provide information about the current state of the harvesting module, e.g., the voltage level and the amount of power and energy extracted. Based on this, the microcontroller can adapt its behavior in order to optimize the transmission rate.

Two different operating frequencies are used for harvesting and communication channels (868 MHz and 433 MHz respectively). On one hand, *harvesting module* is optimized for a constant RF source, so communication signal cannot be effectively used and two separate antennas are required. On the other hand, using two different antennas for harvesting and communication allows optimization of the harvesting antenna to best fit the DC/DC specifications, at the expense of a reasonable increase in area. Using a single antenna would be more complicated and would result in worse harvesting performance, mainly because of the losses introduced by the multiplexing circuitry.

An overview on recent rectenna solutions for generic RF wireless energy transfer applications is available in [8], whereas a comprehensive perspective on RF harvesting techniques specifically devoted to wireless sensor nodes can be found in [3]. In this field of application most solutions concentrate the greatest effort in optimization of the harvesting module, while other studies are proposed to define advanced algorithms to find most efficient transmission rates in complex scenarios. Differently, this work aims at optimizing the full energy chain from the rectenna to power management. Enhanced rectenna designs are proposed in [9] with 40% (simulated) efficiency at −20 dBm input power at 868 MHz, in [10] with 20% (measured) efficiency at −20 dBm input power at 2450 MHz, and in [11], where both solar and RF sources are deployed by a compact structure. Concerning integrated RF-to-DC converters, an interesting solution is presented in [12] with a 65% efficiency at −20 dBm in UHF band and output voltage up to 1.6 V. Another notable solution is presented in [13], where 1.0 V output is obtained at 27 m distance. The nano-power design of an integrated 1 μW – to 5 mW power management circuit is shown in [14]. The DC/DC converter proposed achieves a peak conversion efficiency of 77% and a minimum start-up voltage of 223 mV. The trend for quiescent power consumption of commercial and academic PMIC implementation is shown in [15]. A complete solution with harvesting module and sensing node is proposed in [16], showing an operative range of 5 m with −8 dBm input power. Furthermore, in [6] a simple adaptive solution is adopted, with solely two possible transmission data-rates and a solar cell as harvesting source. Advanced adaptive solutions are studied in [17], for a general-purpose sensor node in a multi-antenna power transfer scenario. These solutions lie on the development of complex recursive algorithms based on the knowledge of the evolution of stored energy in time, which implies the use of additional power-consuming electronics incompatible with micropower scenarios. In fact, experimental results demonstrate just a 5-m operative range. A similar approach is used in [18], with an accurate analysis of harvest-and-use and harvest-store-use schemes. Again, the resulting optimization algorithm requires the development of dedicated energy meters interacting with the node MCU to estimate the correct transmission rate. Both solutions are based on an adaptive estimation of best data-rate accomplished by the MCU on the basis of complex monitoring of available energy. Although DASH7 protocol performs a communication range up to 5 km, it is also widely used for indoor applications and, in combination with RF harvesting, it can provide an optimum solution for enclosed sensors like smart meters placed inside walls or generic structures. Moreover, having two different interfaces for harvesting and communication allows placing the power source regardless the location of the receiving gateway, which can be even far farther away if enough energy is provided.

The proposed solution is a fully autonomous RF energy-harvesting node, where all components (rectenna, DC/DC converter and sensing platform) are jointly optimized in order to obtain the best performances within distances up to 17 m. This work also proposes a micropower-compatible adaptive power management, based on both dedicated circuitry and microcontroller firmware. The introduction of smart voltage supervisors allows the harvesting module to adaptively configure the data transmission period of the node, and to accordingly change the sleep policy. The combination of a nano-power DC/DC converter, smart supervisors and a complex wireless sensor nodes allows operation with negligible input power and state-of-the-art operative distances up to 17 m.

## 2. Materials and Methods

### 2.1. The Harvesting Module

Incident RF power in the standard 868 MHz band (scavenged or intentionally transmitted) is captured from the environment by a rectifying antenna, or rectenna, which converts it into DC power. Due to the unregulated nature of the output voltage and current, and to the strong dependence of the output voltage on the load current, a power management module is also required to regulate, store, and distribute the harvested power to the sensor node.

#### 2.1.1. Rectenna

**Figure 2:**
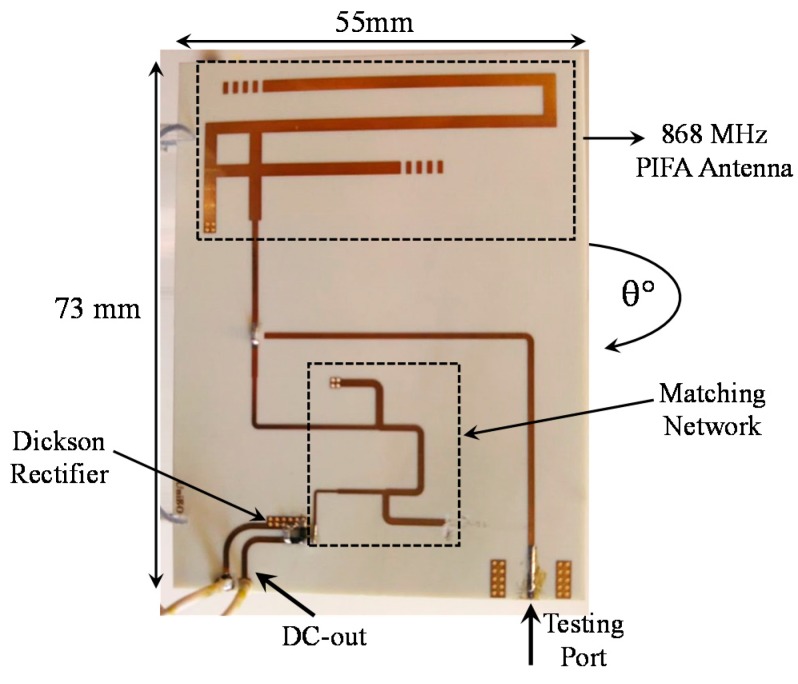
Rectenna prototype with highlighted different sections.

This section describes the rectenna unit we devised and reports on its performance. Figure 2 shows a photo of the discrete-component prototype: it consists of a PIFA (Planar Inverted F Antenna)-like printed antenna which is matched to the RF detector input port by means of a multistage distributed-element matching network.

**Figure 3:**
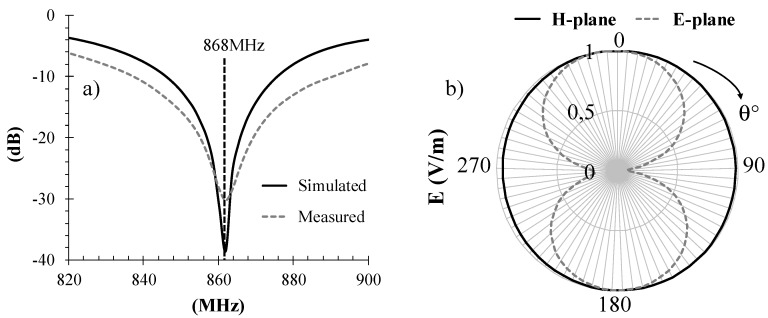
PIFA-like antenna performance: (**a**) reflection coefficient and (**b**) E and H-fieldradiation pattern at 868 MHz.

The first step towards rectenna optimization is antenna design. To satisfy the space constraints of typical sensors for WSN applications [19,20], we selected a compact PIFA-like antenna designed to operate in the 868 MHz band (Figure 2). The topology is similar to that proposed for a completely different type of application [21] in which the target was sensitivity rather than joint maximization of output power and voltage. It consists of two branches in which the lengths have been optimized to tune the antenna to the required resonant frequency and to meet the specifications on radiation efficiency, radiation pattern and input return loss. The overall substrate size of the final design is 73 × 55 mm^2^. The commercial substrate chosen was a Roger 4350B (*ε_r_* = 3.55, tan *δ* = 0.0031, thickness = 1.5 mm). Figure 3a shows the simulated and measured input reflection coefficient of the PIFA antenna. In addition, Figure 3b shows the simulated E- and H-plane at the operating frequency. As expected, the antenna has an omnidirectional radiation pattern in the horizontal plane: the low-directivity behavior is a desirable feature for RF energy harvesting scenarios, where the direction of the incoming power is typically unknown. The corresponding peak gain achieved is 1.4 dBi. All simulations were carried out using a commercial full-wave simulator (*CST Microwave Studio 2016*).

The second step consists in designing the rectifying section, which involves choosing a rectifier topology and a nonlinear design for the rectifier-antenna matching network. As regards the former, single-stage full-wave voltage doubler topology represents the best option for ultra-low power applications, as is well documented in the literature [22,23]. For this reason, a full-wave Dickson rectifier based on low-threshold Skyworks SMS7630-079 Schottky diodes was selected.

**Figure 4:**
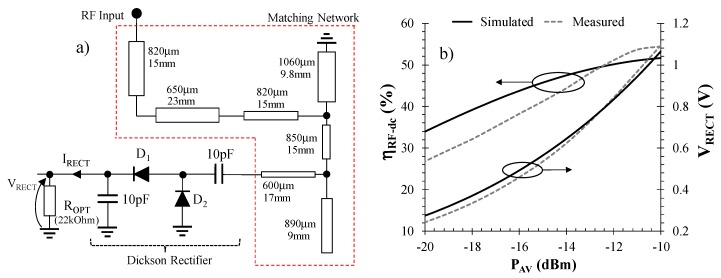
Rectifying section performance: (**a**) matching network and rectifier schematic topologies and (**b**) RF-to-dc conversion efficiency and output dc voltage versus input poweravailable.

Once the rectifier topology is selected, an optimum matching network between the rectifier and the antenna needs to be designed by nonlinear circuit techniques, in order to ensure, throughout the power range available, that maximum RF power enters the rectifier input. The topology chosen is the distributed matching network shown in Figure 4a: it consists of two impedance steps and two stubs, the first being short-circuited to ground whereas the second is open. Impedance steps are employed to guarantee broadband matching between the RF detector (i.e., the rectifying circuit) and the antenna. For the operating frequency (868 MHz) chosen here and for the targeted RF input power, ranging from −20 to −10 dBm, a nonlinear regime is optimized accounting for the dispersive behavior of the linear sub-network (antenna and matching network), represented by a frequency-variable complex reflection coefficient computed by full-wave simulation. The nonlinear model of the diodes is completed with their package model, which is essential for accurate optimization of the entire rectenna at these operating frequencies. Optimization is based on the Harmonic Balance (HB) method and aims at maximizing the RF-to-dc conversion process, through optimization of rectenna efficiency, defined as [24]:

(1) $$\eta_{RF-dc}=\frac{V_{RECT}\;\cdot \;I_{RECT}}{P_{AV}}$$

where *V_RECT_* and *I_RECT_* are the dc components of the rectified voltage and current at the output port when a certain load is applied, while *P_AV_* is the power available at the rectenna location, and represents the maximum power the antenna is able to deliver to the rectifying circuit. Commercial simulator ADS was used for the integrated nonlinear/EM design optimization. Figure 4a shows the circuit layout, with details on the microstrip line dimensions and on the optimum load resistor. Such a rectenna system has been fabricated and the corresponding performance is reported in Figure 4b, where the simulated and measured behavior of the RF-to-dc conversion efficiency, computed as in (1), and the output dc voltage are plotted as a function of the input power (*P_AV_*): the rectenna exhibits a conversion efficiency better than 25% throughout the entire range of low RF power, starting from −20 dBm. In particular, at −18 dBm the efficiency is greater than 30%, corresponding to a dc power of about 6 μW, which represents a practical limit for current state-of-the-art DC/DC converters [25]. At the same time, a dc output voltage greater than 200 mV has been experimentally verified for *P_AV_ =* −20 dBm. All these values were obtained with respect to an optimum dc load of 22 kΩ.

In order to estimate the maximum achievable distance of a sensor equipped with the proposed rectenna from an RF source, the system was tested in a 15 × 15 m^2^ laboratory environment. Experiments took place purposely outside an anechoic chamber in order to account for a more realistic scenario including limited multipath effects; nevertheless measured received power was tolerably close to the theoretical value derived from Friis equation.

**Figure 5:**
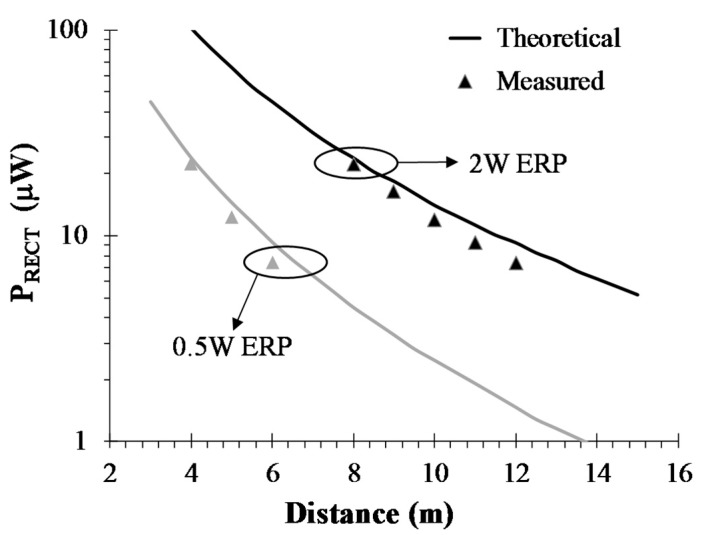
Measured and simulated rectified power for different link distances.

The measurement procedure was carried out in two steps: (i) first, the receiver-to-transmitter distance was set; (ii) then, measurement of the output dc voltage on the rectenna load was carried out. The distances involved in the measurements ranged from 4 to 13 m. The transmitted power was selected following the current regulations for RFID applications [26]: 0.5 W in the 863–870 MHz frequency range and 2 W for a narrow band starting from 869.4 MHz to 869.65 MHz. The output DC voltage on the optimum load R_OPT_ (22 kΩ) was measured by a digital multimeter. This setup lends itself to effective experimental verification of the actual behavior of the entire rectenna, i.e., the antenna acting as the power source of the rectifier. In order to create a reference comparison with the expected simulated results, the link adopted was first modeled by a Friis equation and the actual rectified power (*P_RECT_*) was straight forwardly computed by the following equation:

(2) $$P_{RECT}=P_{TX}\cdot G_{TX}\cdot G_{RX}\cdot {\left(\frac{\lambda }{4\pi \cdot D}\right)}^{2}\cdot \eta_{RF-dc}$$

where *P_TX_* is the power transmitted at the remote location (the power exciting the transmitting antenna), *G_TX_* and *G_RX_* are the gains of the receiving and transmitting antennas, respectively, *D* is the link distance, λ is the wavelength at the operating frequency. It is noteworthy that experimental and theoretical analyses may be carried out considering the maximum gain direction, for both the transmitting and receiving antennas. As the transmitting antenna, we adopted a commercial logperiodic antenna (*PCB VA5JVB*), with *G_TX_* = 6 dBi. As mentioned before, the receiving antenna gain is *G_RX_* = 1.4 dBi. Figure 5 reports the rectified dc power versus distance, for two different ERP levels. Considering a minimum required power of 6 μW for the dc-dc converter [27], it can be concluded that the rectenna is able to operate at 6-m distance for the 0.5-W-ERP experiment, and up to 12-m distance for the 2-W-ERP. It is noteworthy that the models used during rectenna optimization for both the dispersive linear sub-circuit and the nonlinear devices allowed one to obtain predicted performances that were in very good agreement with the experimental one over a wide range of transmitted RF power, as can be observed in the plots of Figure 5 where the measured and predicted rectenna output dc power are compared over a number of RF source-rectenna distances.

#### 2.1.2. DC/DC Converter

**Figure 6:**
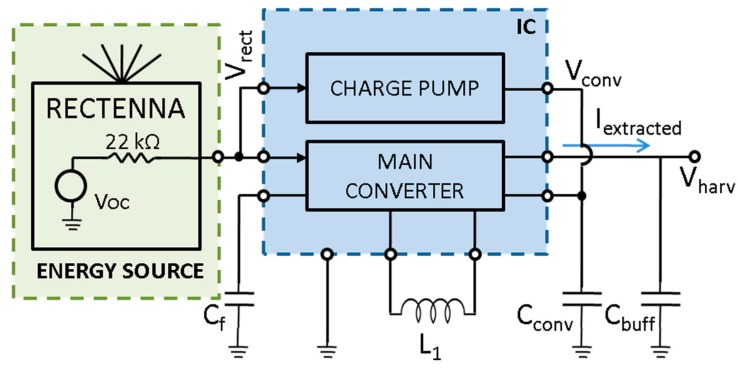
DC/DC low-power converter block diagram.

The DC/DC converter used in the system features an ultra-low power buck-boost converter designed in STMicroelectronics 0.32 μm CMOS microelectronic technology [14]. The overall architecture can be divided into two main blocks: a start-up circuit, which allows for IC bootstrap with RF input sources typically providing low voltages, and the main DC/DC converter which also provides a fractional open-circuit voltage (FOCV) MPPT algorithm in order to adapt to the best power transfer condition. The IC dynamically decides whether to route power to the load or to a small self-supply capacitor C_conv_: this achieves very fast activation times even in the presence of large buffer capacitors at the load output port. When C_conv_ is sufficiently charged, all power is routed to the load. Should C_conv_ get excessively discharged, all power is routed here to replenish it before the IC fails. A block diagram of the circuit IC is reported in Figure 6.

The start-up module consists of a 16-stage charge pump implemented with low-threshold MOSFETs and driven by an internal oscillator. A minimum voltage of approximately 250 mV from V_rect_ is required to keep it operating. During the start-up phase, when the input voltage (i.e., the rectenna output voltage) approaches 250 mV, the charge pump circuit starts working, and the output voltage on the self-supply capacitor C_conv_ is boosted. However, internal devices are not fully switched on until the output voltage gets to 600 mV, so that the output charging rate is initially limited by the sub-threshold state of the system. As soon as the generated voltage reaches 0.6 V the start-up circuit becomes fully operational and the charge pump improves its charging rate until the output exceeds 1.36 V, which is the minimum operating voltage of the main DC/DC converter. At this point the charge pump is disabled and power conversion occurs through the buck-boost DC/DC converter. Although the overall efficiency of the start-up stage settles between 5% and 15%, its sole purpose is to initially bootstrap the main DC/DC converter, so that its impact on operative efficiency can be considered negligible.

Once the 1.36 V threshold is reached, an in-rush current of about 11 μA is absorbed from the energy source by the module for a short time to complete bootstrapping the converter functionalities. After this, the module can be sustained with an input power of just 935 nW showing a quiescent current of 121 nA. The different functional modes are summarized with the corresponding supply voltages in Table 1. It is worth recalling that these values refer to the voltage on the self-supply capacitor V_conv_. Another aspect worth consideration is the high output resistance of the rectenna, which causes significant voltage drops on its output node as the current increases.

In order to extract the maximum power from the source, the IC adopts an FOCV MPPT technique. The input source is kept at 50% of the open-circuit voltage of the rectenna, which actually represents the maximum power transfer condition for the system. The open-circuit voltage is sampled for 2 μs every 8 conversion cycles of the DC/DC converter. The buck-boost DC/DC converter operates in discontinuous current conduction mode, and is switched when the source voltage crosses the reference MPPT voltage. The overall efficiency is highly affected by source impedance and open-circuit voltage.

**Figure 7:**
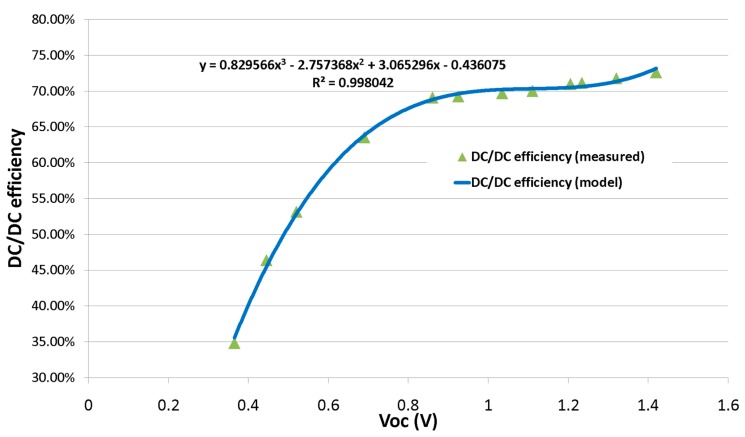
DC/DC efficiency vs. open circuit input voltage.

Thereafter, a full analysis was performed with the aim of obtaining an exhaustive characterization of the DC/DC converter in the specific running conditions of the system proposed. An equivalent model of the rectenna described in Section 2.1.1 was extracted from the static voltage-current characteristics and used for characterization. This consists in a DC voltage source (V_OC_) with a 22 kΩ series resistance. The output voltage V_harv_ of the DC/DC is set at 2.3 V, which is the chosen maximum operating voltage of the sensing node as described in Section 2.3. During operation, the variations on V_harv_ will be limited to a few hundred mV as the worst case. A 10 MΩ load was connected to V_harv_. Results show that when the open-circuit voltage of the rectenna V_OC_ ranges from 0.32 V to 1.4 V, the related efficiency grows from 35% to 73%, with a plateau reached early on at 0.8 V. The whole characterization is reported in Figure 7.

**Figure 8:**
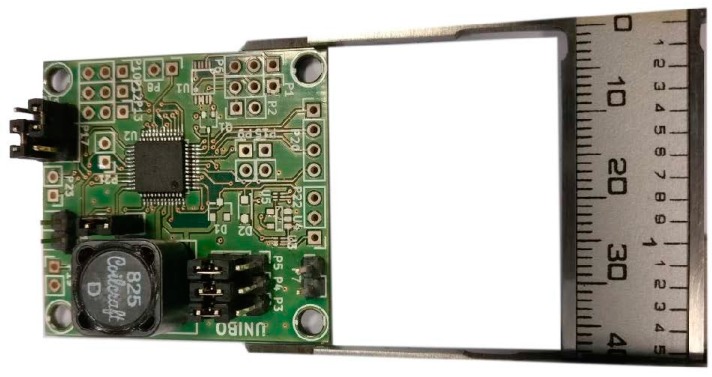
Harvesting module board.

With respect to Figure 4b, VRECT will be kept by the IC at approximately half of VOC, as the DC/DC converter works to maintain a state of maximum power transfer by providing an input impedance equal to the source impedance (22 kΩ in this case). Moreover, the extracted model was considered valid throughout the operative range of the system. Figure 8 shows the dedicated board containing the DC/DC converter along with its subsidiary components.

### 2.2. The Active Node

**Figure 9:**
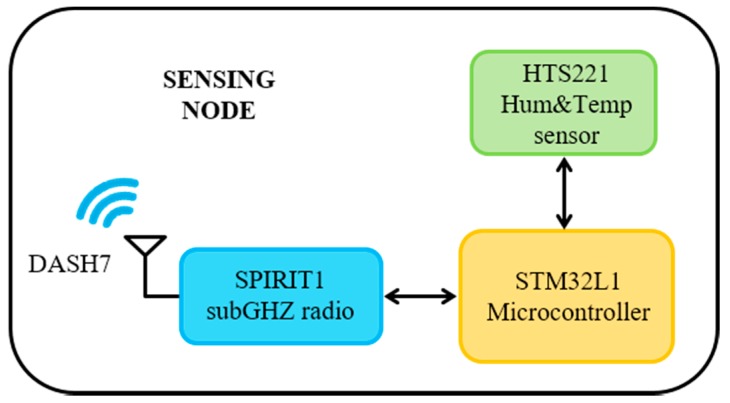
Block diagram of the sensing node.

The sensing and communication node, whose internal architecture is shown in Figure 9, includes a temperature and relative humidity sensor, a low-power microcontroller and a sub-GHz radio device for data communication. STMicroelectronics HTS221 is an ultra-compact sensor based on a planar capacitance technology that integrates humidity and temperature sensing with a mixed signal ASIC to provide data measured through standard digital serial interfaces. The ultra-low power STMicroelectronics STM32L1 microcontroller, based on the ARM Cortex-M3 core, interfaces with the sensor for data acquisition and manages communication with the STMicroelectronics SPIRIT1 module, a low-power sub-GHz RF transceiver.

Wireless communication is based on DASH7 [28], an open source Wireless Sensor and Actuator Network (WSAN) protocol; the microcontroller implements OpenTag, a DASH7 protocol stack and a minimal Real-Time Operating System (RTOS) designed to be light and compact and targeted to run on resource-constrained microcontrollers.

The DASH7 network architecture has a star structure where all the nodes, which are typically low-power devices able to transmit and receive data, communicate only with a gateway that is never offline and connects the DASH7 network to other networks and to the web.

DASH7 supports two communication models: pull and push [29]. The pull model consists of a request-response mechanism initiated by the gateway; it uses an advertising protocol for rapid ad-hoc node synchronization before sending an addressed request to a node and waiting for the response [29]. The data transfer to the gateway initiated by the nodes is based on the push model (e.g., beaconing); this approach is implemented as an automated message or beacon that is sent at specific time intervals.

In this work, the beaconing approach is used to send the temperature and humidity data from the sensor node to the gateway; this method is the least power hungry and the node can send the message as soon as the harvesting module provides enough power to power-up the node or wake up it from a deep sleep state.

The node can be supplied with a voltage ranging from 1.8 V to 3.6 V, and is programmed to perform two different stop policies: *off-mode* and *standby-mode*. The policy is selected by the harvesting module, as will be explained in the following section.

**Figure 10:**
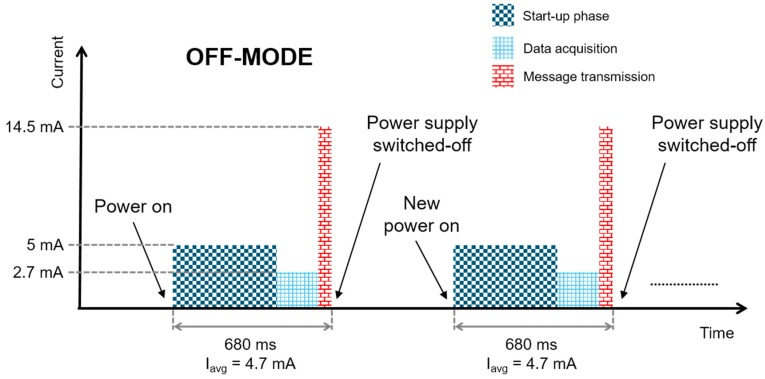
Schematic, not to scale, of the node current consumption in off-mode; the average charge consumption in each activation phase is Q=I*T=4.7 mA*680 ms=3.196 mC.

**Figure 11:**
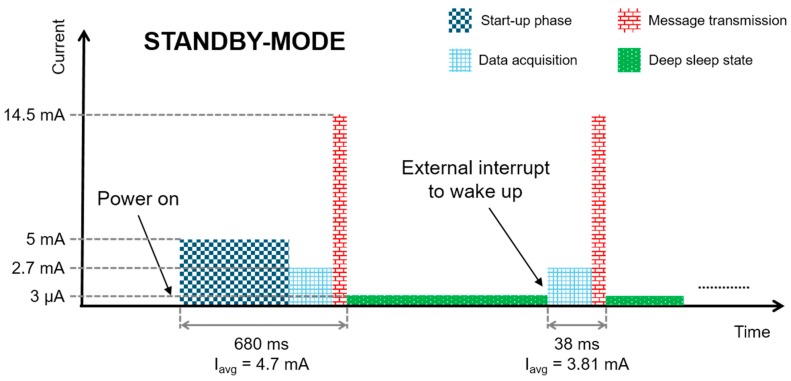
Schematic, not to scale, of the node current consumption in *standby-mode*; the average charge consumption in each activation phase following the power on phase is Q=I*T=3.81 mA*38 ms ~ 145 μC.

As can be seen in Figure 10 and Figure 11, the two modes behave in the same way at power on, when, after the start-up phase, the node acquires data from the sensor and transmits them to the gateway. Afterwards, if *off-mode* is selected, the node generates a signal for the harvesting module to inform it that the power supply can be switched-off. When the power supply is once more provided, the node will perform a new start-up phase, data acquisition and message transmission. This phase is associated with a significant energy overhead. Figure 10 shows a schematic of the current consumption of the node and its behavior in *off-mode* indicating the average current in each of the three phases (start-up, data acquisition and transmission); in all each activation phase lasts 680 ms and the average current consumption over this time is 4.7 mA at 1.9 V.

On the contrary, if the *standby-mode* is selected, after the first start-up phase and message transmission, the node informs the harvesting module that transmission is completed and that it is entering a deep sleep state in which its current consumption is 3 μA. The node then waits for an external interrupt, generated by the harvesting module, to wake up and perform a new data acquisition and message transmission. In *standby-mode*, therefore, the power supply is never switched-off, and the overhead consists in constant power consumption. The schematic of the node’s current consumption and its behavior in *standby-mode* is shown in Figure 11. In this configuration, with the exception of the first power-on in which the current consumption is the same as each activation in *off-mode*, the start-up phase does not have to be repeated for each activation phase because the power supply is never switched-off. As can be seen in Figure 11, in *standby-mode* each activation phase following the first one lasts 38 ms, and the average current consumption during this time is 3.81 mA at 1.9 V. In *standby-mode*, the charge consumption for each data acquisition and message transmission is much less than in *off-mode*, but a current of 3 μA has to be guaranteed to keep the node alive in the deep sleep state.

The selection between *off-mode* and *standby-mode* has a significant impact on the power budget. The *off-mode* is associated with a constant energy overhead, consisting in the energy consumed for starting the system. By contrast, the *standby-mode* is associated with a constant power overhead. Hence, which of the two modes costs less will depend on the frequency of activation. For frequent activation, the stand-by mode consumes less energy. Furthermore, the frequency of activations strictly depends on the harvested power.

**Figure 12:**
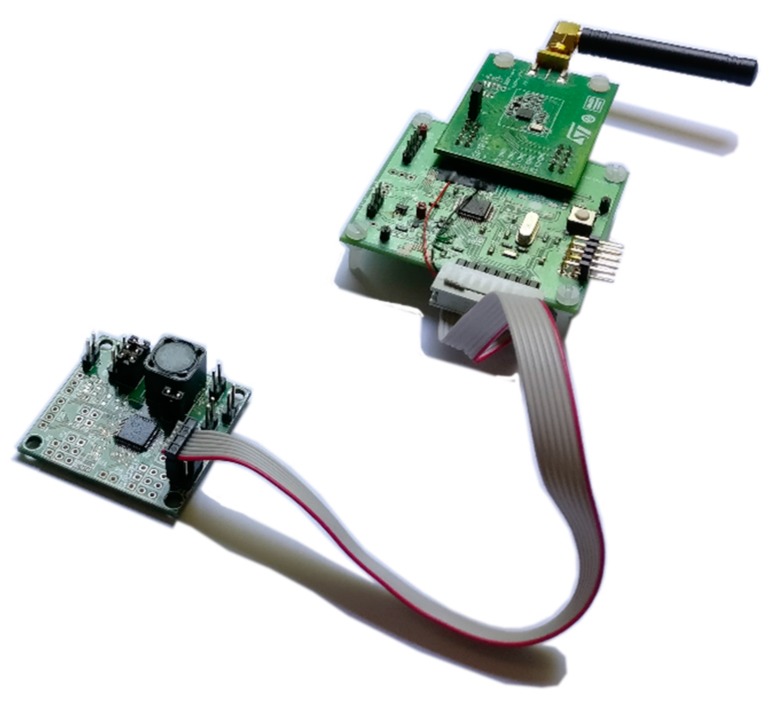
Sensing node and harvesting module boards.

Figure 12 shows the sensor node connected to the harvesting module describer in Section 2.1.

### 2.3. Policies for Power Optimization

**Figure 13:**
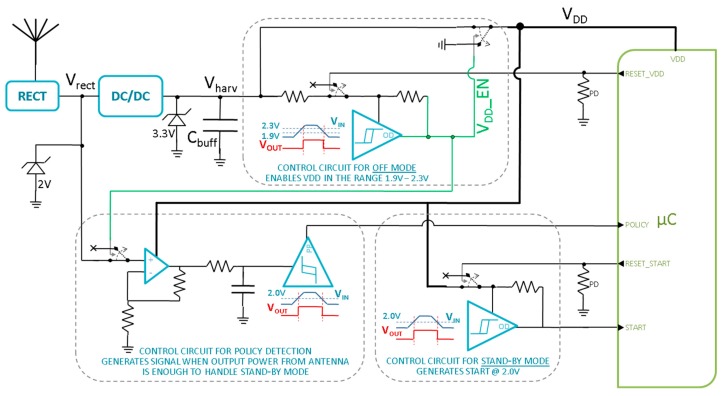
Schematic of the harvesting module and its interface with the active node.

In this section, the proposed adaptive feature is described in detail, as well as the architectural choices and dimensioning of control circuits shown in Figure 13. The interface between the harvesting module and the active node is implemented through four control signals connected to the microcontroller (*Policy*, *Reset_V_DD_*, *Start* and *Reset_Start*), besides the ground and positive supply (*V_DD_*).

The *Policy* signal selects one of the two stop policies of the active node (*off-mode* and *standby-mode*).

The first factor to be analyzed is the buffer capacitance *C_buff_*, which plays the role of energy storage needed by the system to offset the total power request of the node during its active phase. Such requests are summarized in Table 2, which integrates the current absorbed by the node when switching into active mode from each of the other two modes.

In order to have a voltage drop of about 0.4 V, the resulting *C_buff_* must be at least

(3) $$C_{buff}=\frac{Charge\left(OFF\right)}{∆V}=\frac{3196\;\mu C}{0.4\;V}=7990\;\mu F≅8\;\mathrm{mF}$$

where the voltage drop was chosen as a trade-off between system power consumption and wake-up time. In this instance, a lower bound of 1.9 V was chosen by considering a 100 mV margin to the minimum 1.8 V supply voltage required by the node to operate, while the upper bound was chosen by considering that a lower voltage implies lower power consumption, though at the same time it requires a larger buffer capacitance which causes a longer start-up time when the system has to be booted for the first time. Hence a maximum operating voltage of 2.3 V was chosen and, as a result, an 8 mF *C_buff_* must be considered.

Three control circuits are necessary to generate the correct control signals for the micro-controller (Figure 13).

The first one is the *Control circuit for OFF mode*. This block has the task of monitoring the voltage across *C_buff_* capacitance voltage and consequently to provide power supply to the sensing node only when voltage is in the acceptable range (1.9 V–2.3 V). In this way, the sensing module is only switched on when *V_DD_* exceeds 2.3 V, i.e., when the buffer capacitance has sufficient energy to sustain at least one complete data transmission. Likewise, power supply is taken off as soon as *V_DD_* drops below 1.9 V, as the sensing module cannot work at lower voltages. During *off-mode*, a reset signal (*Reset_V_DD_*) must be issued by the microcontroller at the end of each transmission stage, so that power supply is simultaneously turned off. This behavior ensures that the next transmission will only be held when the buffer capacitance is fully recharged, i.e., when it reaches 2.3 V again. A NCP303 low-power voltage supervisor was used to implement the block, along with two AS11P2 analog switches and two resistors in order to obtain the desired hysteresis, respectively 2.4 MΩ and 500 kΩ. The total quiescent current of the block is 500 nA, mostly due to the NCP303 supervisor.

The second one is the *Control circuit for STANDBY mode*. This block has the task of monitoring the capacitance voltage and consequently providing the *start* signal only when voltage is above 2.0 V. During *standby-mode*, the sensing node is always powered, and subsequent transmissions are regulated by the *start* signal, so that it must only be issued when the buffer capacitance is storing enough energy to sustain transmission. Actually, the expected voltage drop due to a single transmission in *standby-node* is a mere 18 mV as explained in following equation

(4) $$∆V\left(STANDBY\right)=\frac{Charge\left(STANDBY\right)}{C_{buff}}=\frac{145\;\mu C}{7990\;\mu F}=18\;\mathrm{mV}$$

so a threshold voltage of 1.9 V + *∆V* = 1.918 V could be enough to sustain standby-mode, but a safer margin of 2.0 V − 1.9 V = 100 mV was chosen for this condition. A reset signal (*Reset_Start*) from the microcontroller is also requested at the end of each transmission in order to force the *start* signal to a low level, even if the related capacitance voltage drop has not been sufficient to trigger the voltage supervisor. This happens because the microcontroller is sensitive to a positive *start* signal edge, so that a low-to-high transaction is always required to awaken the sensing node. An NCP303 low-power voltage supervisor was used to implement the block, along with one AS11P2 analog switch and a pull-up resistor of 2.4 MΩ. The total quiescent current of the block is 400 nA, mostly due to the NCP303 supervisor.

The ability to dynamically select whether to operate in *off-mode* or *standby-mode* is a crucial aspect of the solution presented, as it enables the system to fully exploit the available environmental energy under any circumstance without human intervention. In both cases the minimum transmission period is obtained, as the microcontroller is either supplied (*off-mode*) or awoken (*standby-mode*) as soon as the buffer capacitance has recovered the energy lost, but depending on the variable amount of extracted energy, one solution can be less advantageous than the other, if not altogether unfeasible. Transmission Period (*TP*) is defined as the time interval between two consecutive transmissions and can be calculated as:

(5) $$TP=\frac{Q_{needed}}{\left(I_{extracted}-Iq_{harv}-Iq_{node}\right)}$$

where *Q_needed_* is the charge lost during each transmission, *I_extracted_* is the current provided by the DC/DC converter, *I_qharv_* is the quiescent current of the harvesting module that is directly supplied by V_HARV_ while *I_qnode_* is the quiescent current of the rest of the system supplied by *V_DD_*. These values differ between the two operative modes, and are summarized in Table 3.

The most significant difference is that in *off-mode* the charge needed is significantly higher than in *standby-mode*, but the total quiescent current is only 500 nA, since all the other modules are switched off during recharge periods. As a consequence, in *off-mode* nearly all the current from the power converter can be used to recharge the buffer capacitance.

**Figure 14:**
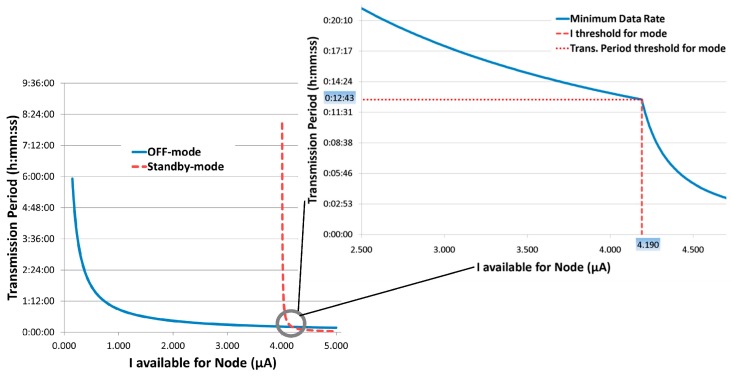
Transmission period vs. Extracted currents in different operating modes.

Thus, this last mode proves more suitable when the extracted current is relatively low, whereas the *standby-mode* becomes more cost effective at higher extracted currents, when its lower value of activation charge becomes predominant. A detailed graph of transmission data rates versus extracted currents is shown in Figure 14, analytically obtained through Equation (5).

The available current is defined as the effective current that can be used by the active part of the system or, in other words, the whole extracted current subtracted by *I_qharv_*, which is always drawn from the DC/DC converter (*I_available_* = *I_extracted_* − *I_qhar_*_v_). As shown in the graphs, as the available current rises above 4.190 μA, *standby-mode* becomes the best choice in terms of minimum data rates, and the related threshold value is about 13 min.

With this assumption, the *Control circuit for policy detection* has the task of correctly identifying whether the current available is either above or below the threshold value of 4.190 μA, and communicating this to the microcontroller through the *policy* signal. Since the correct relationship between the power received by the rectenna and the power extracted by the DC/DC has been fully investigated (see details in Results section), the least power-consuming solution is to simply monitor the average voltage at the rectenna output. This value is solely related to the extracted current through the efficiencies of the interposed stages, and experimental results show that a value ≥585 mV for V_RECT_ is required to obtain an available current of 4.190 μA. Since the low-power voltage supervisor NCP303 has a threshold value of 2 V, a dedicated low-power voltage amplifier with a 2 V0.585 V=3.42 gain was added, composed of an LPV521 op-amp and two resistors of 20 MΩ and 4 MΩ. Moreover, a simple RC filter was inserted with τ = 100 ms, in order to reject the periodic fluctuations caused by the FOCV algorithm of the DC/DC converter, which actually disconnect the rectenna for about 4 μs every 8 extraction cycles. The overall quiescent current of the block is 600 nA, as already reported in Table 3.

## 3. Results

The fully autonomous DASH7 IoT sensor node with RF harvesting capability was characterized experimentally. Moreover, we tested the capabilities of implementing the adaptive behavior.

The available energy is mainly affected by two factors: the transmission power and the distance between transmission and receiving antennas. For the former, two different transmitter powers were selected to be compliant with the RFID standard [26], respectively 0.5 W ERP and 2.0 W ERP. Then, a full analysis with respect to values of distance was performed. Starting from experimental results for the rectenna (Figure 4) and DC/DC (Figure 7), a precise analytic model of the harvesting module was extracted, and this provides the power extracted by the DC/DC converted as a function of RF source power and node distance. The related results are presented in Section 3.1. Furthermore, model reliability was proved by two experiments accomplished with a regulated RF source placed at a defined distance from the harvesting module (logperiodic directive antenna “*PCB VA5JVB*”, with *G_TX_* = 6 dBi).

Combining the harvesting analytic model described in Section 3.1 with Equation (5) and the measured currents reported in Table 3, yielded a detailed estimate of startup times and transmission periods as reported in Section 3.2. The foregoing results are summarized in Section 3.3.

Section 3.4 presents the experimental results of the sensor node, while Section 3.5 describes the tests performed on voltage supervisors.

### 3.1. Harvesting Module Results

**Figure 15:**
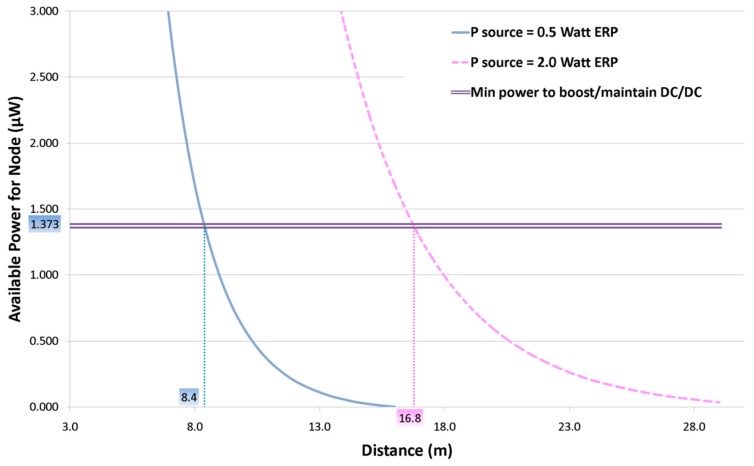
Power extracted vs. Node distance.

First of all, a specific graph reporting the available power versus node distance is presented in Figure 15. The available power is the total power extracted from the DC/DC converter. It also accounts for losses on the capacitor and on instrumentation.

The graph shows two different curves for 0.5 W ERP and 2.0 W ERP, which intersect at threshold values. The value of 1.373 μW is the threshold value at which the system presented can be considered self-sustained, as it can successfully supply the minimum actual load of the system, which is the control circuit for *off-mode* described in Figure 13.

The far from inconsiderable result obtained is that with a transmitted power of just 0.5 W ERP the system presented can start at 8.4 m, while with 2.0 W ERP the distance can rise up to 16.8 m.

Minimum distance of 8.4 m with 0.5 W ERP transmitted power was verified through a dedicated experiment.

### 3.2. Startup/Transmission Period Results

When the system starts from a completely discharged situation, an initial startup time is required to charge the 8-mF buffer capacitance whose value depends on the harvested current.

**Figure 16:**
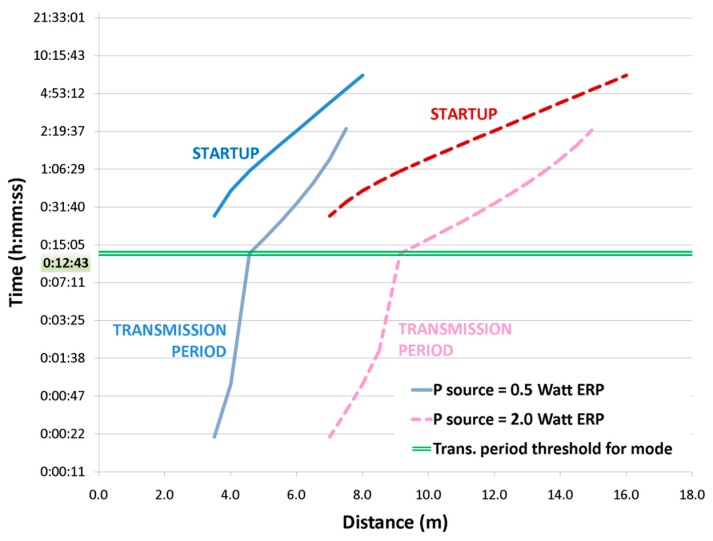
Transmission data rate and policy relationship with node distance.

Once booted, the node is automatically configured to operate in the best operating mode, providing the shortest transmission period. A detailed graph of startup and transmission times is reported in Figure 16.

Analytic results show that the minimum transmission period is about 21 s, corresponding to an extracted power of 26.5 μW obtained with an antenna open circuit voltage of 1.650 V. Similarly, the operating mode is switched from *off-mode* to *standby-mode* according to Figure 13 when the extracted power is 11.011 μW (open circuit voltage 1.170 V), with a period of 12′43″.

### 3.3. Power/Transmission Period Combined Results

**Figure 17:**
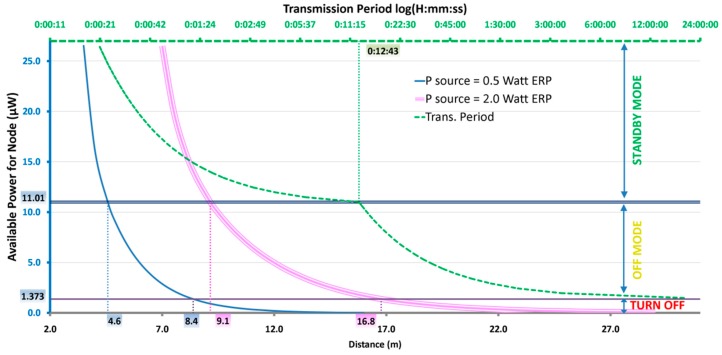
Combined graph showing the relationship between distance, extracted power and transmission periods.

Figure 17 shows the relationship between node distance, power extracted from DC/DC and obtainable transmission periods. Starting from distance (bottom horizontal axis), the single blue line represents extracted power when the source is 0.5 W ERP, the triple pink line is extracted power when the source is 2.0 W ERP. Once the available power is known, the obtainable period can be found on the dotted green line, whose values are reported along the top horizontal axis. Moreover, three operative regions of extracted power are identifiable: a *turn off* region where the node cannot be switched on, an *off-mode* operating region and a *standby-mode* one.

Table 4 recaps the threshold values shown in Figure 17.

### 3.4. Sensor Node Results

**Figure 18:**
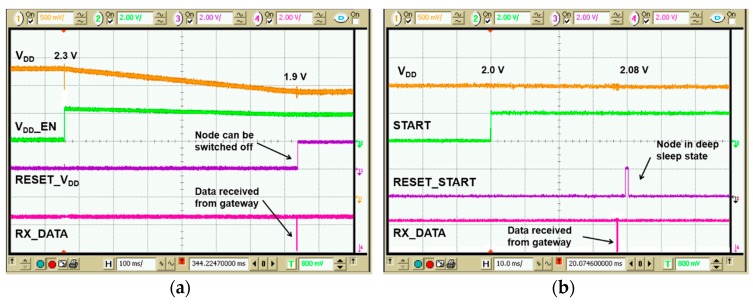
Voltage drop and control signals of micro-controller in *off-mode* (**a**) and *standby-mode* (**b**).

The effective operation of the DASH7 node was tested, in terms of power consumption, transmission capability and adaptive data rate. Figure 18a shows waveforms of signals related to *off-mode operation*. Supply *V_DD_* decreases as expected from 2.3 V to 1.9 V over a time interval of about 680 ms, which correspond to a single transmission. With reference to Figure 10, this time accounts for the microcontroller start-up phase, the data acquisition and finally data transmission. The reset signal (*Reset_V_DD_*) is activated at the end of each transmission. Similarly, in Figure 18b the control signals for *standby-mode* are shown. Voltage drop is about 20 mV as expected, while in this mode the *Reset_Start* signal is activated instead of *Reset_V_DD_* at the end of transmission.

**Figure 19:**
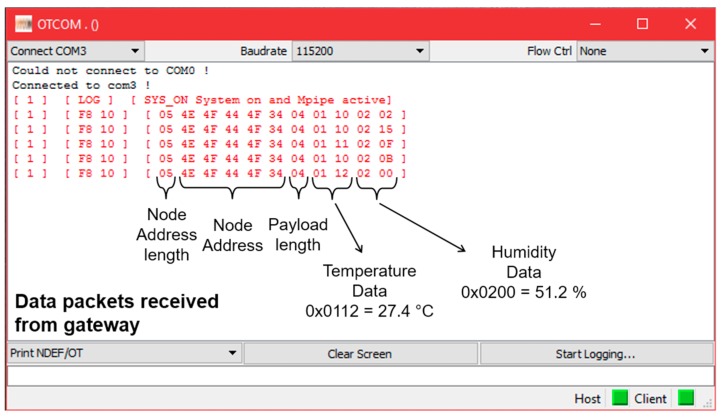
Data received format.

Transmitted data are then received by a dedicated gateway, which displays data directly onto a PC terminal. Figure 19 shows the received data bytes with different sensor values.

### 3.5. Voltage Supervisor and Policy Selection Results

**Figure 20:**
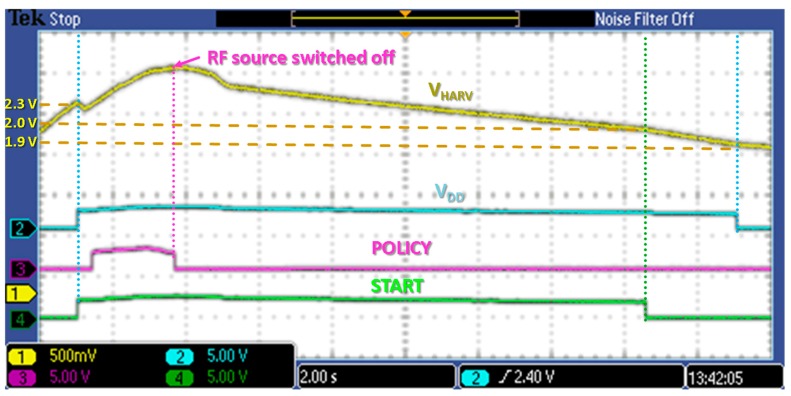
Power optimization circuits and policy detection waveforms.

Finally, power optimization circuits for policy selection were tested. In Figure 20 control signals behavior can be observed with respect to DC/DC output voltage V_HARV_. Considering signals reported in Figure 13, V_HARV_ is initially below the activation threshold of 2.3 V, so all other signals are disabled. As soon as V_HARV_ exceeds 2.3 V, the node can be enabled so *V_DD_* (=V_HARV_) is provided, and other supervisors are supplied through *V_DD_* too. Start signal is high, as V_HARV_ is above 2.0 V, while policy is also high as V_OC_ is kept above 1.170 V in this stage (see Table 4). As RF source is switched off, the buffer voltage V_HARV_ begins to decrease, and policy is immediately forced low. Then start signal goes low when V_HARV_ falls below 2.0 V as expected, while supply voltage *V_DD_* is disconnected when V_HARV_ goes below 1.9 V.

## 4. Conclusions

In this work we proposed an autonomous battery-less sensor node with RF harvesting capability in the 868 MHz UHF band. Temperature and humidity data are acquired and transmitted using a standard DASH7 protocol, which allows the node to be compatible with IoT infrastructure.

An optimized harvesting module, combining a dedicated rectifying antenna and an ultra-low power DC/DC converter with MPPT, is used to obtain considerable results at long distances. Besides that, innovative solutions for adaptive behavior are introduced. In particular, by monitoring the rectifying antenna open-circuit voltage, a dedicated low-power management circuitry can select two different operating modes of the sensing node. Furthermore, in both cases, the minimum transmission period is achieved accordingly to the available RF power through the power management module. The node achieves operation with standard wireless protocol in micropower condition.

As a result, the node is able to operate with an antenna open-circuit voltage of solely 520 mV, corresponding to 12.3 μW input power. Consequently, with a RF source of 2.0 W ERP, the node can be turned on up to 16.8 m distance.

## Figures and Tables

**Table 1:** DC/DC converter functional modes.

**V_rect_**	0 V–0.250 V	0.250 V–1.6 V		
**V_conv_**		0 V–0.600 V	0.600 V–1.36 V	>1.36 V
**mode**	**Switched off**	**Charge pump** (limited efficiency)	**Charge pump** (fully functional)	**DC/DC converter fully functional** • 121 nA quiescent current • 935 nW minimum input power
				**11 μA in-rush current from source needed** to bootstrap the DC/DC converter

**Table 2:** Charge requests of node during active states according to mode.

Mode	Charge (μC)	Operating Voltage (V)
OFF	3196	2.3–1.9
STANDBY	145	~2

**Table 3:** Quiescent currents of harvesting module and sensor node during recharge phase.

	Q_needed_(µC)	*I_qharv_*(µA)	*I_qnode_*(µA)		
		Control circuit OFF-mode	Control circuit STANDBY-mode	Control circuit for Policy detection	Sensor node
OFF	**3196**	**0.5**	switched-off	switched-off	switched-off
			**switched-off**		
		**0.5**			
STANDBY	**145**	**0.5**	0.4	0.6	3
			**4**		
		**4.5**			

**Table 4:** Minimum distances in the three operating conditions.

	Antenna V_oc_ (V)	Extracted Power (μW)	Transmission Period	Distance (m)	
				0.5 W ERP	2.0 W ERP
Switch-on	0.490	1.373	→∞	8.4	16.8
Policy Threshold	1.170	11.011	0:12:43	4.6	9.1
Min. Trans Period	1.650	26.531	0:00:21	3.5	7.0
